# Parents' experiences and satisfaction with care during the birth of their very preterm baby: a qualitative study

**DOI:** 10.1111/1471-0528.12104

**Published:** 2013-01-04

**Authors:** A Sawyer, H Rabe, J Abbott, G Gyte, L Duley, S Ayers, on behalf of ‘The Very Preterm Birth Qualitative Collaborative Group’

**Affiliations:** a School of Psychology, University of SussexBrighton, UK; b Academic Department of Paediatrics, Brighton and Sussex University Hospitals Trust, Royal Alexandra Children's HospitalBrighton, UK; c Bliss (The Special Care Baby Charity)London, UK; d National Childbirth TrustLondon, UK; e Nottingham Clinical Trials Unit, University of NottinghamUK; f School of Health Sciences, City University LondonLondon, UK

**Keywords:** Care, experience, preterm birth, qualitative, satisfaction

## Abstract

**Objective:**

To assess parents' experiences and satisfaction with care during very preterm birth and to identify domains associated with positive and negative experiences of care.

**Design:**

Qualitative study using semi-structured interviews.

**Setting:**

Three neonatal units in tertiary care hospitals in South-East England.

**Population:**

Thirty-two mothers and seven fathers who had a baby born before 32 weeks of gestation and spoke English well.

**Methods:**

Semi-structured interviews were conducted. Results were analysed using thematic analysis.

**Main outcome measures:**

Participants' experiences and satisfaction with care during the birth of their preterm baby.

**Results:**

Overall, 80% of participants were extremely satisfied with the care during the birth of their preterm baby, seven were generally satisfied but felt some things could be improved and one was dissatisfied. Four key determinants of experiences of care were identified: staff professionalism, which included information and explanation, being calm in a crisis, appearing confident and in control, and conversely not listening to the woman; staff empathy, which included caring and emotional support, and encouragement and reassurance; involvement of the father; and birth environment.

**Conclusions:**

Although the determinants of experiences of care are generally consistent with previous research on term births, unique factors to preterm birth were identified. These were the importance of the staff appearing calm during the birth, and the staff portraying confidence and taking control during the birth. Women valued being listened to, and both they and their partners valued staff helping fathers to feel involved during the birth.

## Introduction

Preterm birth is the most important single determinant of adverse outcome in terms of survival, quality of life, psychosocial and emotional impact on the family, and costs for health services. The highest mortality and morbidity occurs in babies born very preterm, defined as delivery before 32 weeks of gestation.^1^ Although only 1.4% of UK births are very preterm, they account for 51% of infant deaths.^2^ The birth of a very preterm baby is often an extremely stressful and traumatic time for parents.^3^–^6^ The birth may have been rapid and unexpected, and parents and baby are usually separated at birth as the baby is taken to the neonatal unit.

Women's views and experiences of maternity services, especially care during labour and childbirth, are increasingly important to healthcare providers, administrators, and policy makers, and can influence decisions about the organisation and provision of services.^7^,^8^ Satisfaction with care during childbirth is also related to the health and wellbeing of the mother and her baby. For example, dissatisfaction is associated with poorer postnatal psychological adjustment, a higher rate of future abortions, preference for a caesarean section, more negative feelings towards the infant, and breast-feeding problems.^9^–^11^ The concept of satisfaction with medical care has been criticised as being complex and often poorly defined.^12^ A useful definition is that a person's satisfaction is a ‘personal evaluation of healthcare services and providers’.^13^^.^

There has been little research into parents' experiences and satisfaction with care during very preterm birth. The aims of this study were to explore parents' experiences and satisfaction with their care during very preterm birth, and to identify the domains associated with positive and negative experiences of care.

## Methods

This qualitative study used semi-structured interviews. Recruitment took place between June 2011 and November 2011 at three tertiary care centres in South-East England. Ethics approval was obtained from the Kent NHS Research Ethics Committee.

Parents were eligible if they had a baby born before 32 weeks of gestation in the previous 6 months and spoke English well. They were also eligible if only one member of the couple wanted to take part or if they were single. Parents of babies who died were also included. Women were not approached if the attending clinicians considered that they were too unwell to take part.

Information about the study was available to parents in the neonatal units. Eligible parents were then invited to participate by responding to a letter of invitation. This was either posted or given to parents if they had been on the neonatal unit for longer than 2 weeks. Parents returned a card indicating their willingness to participate, and the study researcher then contacted them to discuss the study and arrange the interview. Before the start of the interview, participants were given the opportunity to ask questions, and gave written informed consent. All interviews were carried out by the first author (A.S.), who is a psychologist with experience of interviewing women in the perinatal period. Parents were informed that the interviewer was not associated with the hospital so as to encourage open and honest responses. Parents were offered the choice of an interview in a quiet room in the hospital or at their home. Interviews lasted approximately 45 minutes. They were recorded and then transcribed with all identifying information removed. Data collection ended when no new information emerged from the interviews and data saturation had been achieved.

### Materials

The interview schedule consisted of 10 open-ended questions that were used as a guide to explore parents' experiences and satisfaction with care during the birth (Box 1). Three additional questions asked about parents' first experiences with their baby (these questions were analysed separately and will be reported in another paper). The interviewer had the freedom to probe the interviewee to elaborate on the original response or to follow a line of inquiry introduced by the interviewee. Cues and prompts were also used by the researcher to allow the interviewee to discuss the topic further. A questionnaire was used to collect sociodemographic information, and obstetric and neonatal information was extracted from the medical records.

**Box 1. Interview questions**

- Can you describe what led up to your baby's birth?
- Can you describe what happened during the birth of your baby?
- Can you describe what happened immediately after the birth of your baby?
- Can you tell me about your experience of the care you received at birth and immediately after your baby was born?
- Was there anything particular about the care you received (at birth and immediately after your baby was born) that you were happy with?
- Was there anything particular about the care you received (at birth and immediately after your baby was born) that you were unhappy with?
- What aspects of your care (at birth and immediately after your baby was born) were the most important in terms of your level of satisfaction?
- Overall, how satisfied would you say you were with the initial care that you received?
- Do you have any suggestions about how immediate care at birth could be improved?
- Are there any other issues I haven't brought up that you feel are important and you want to talk through?

### Data analysis of interviews

Qualitative analysis of the transcripts used inductive thematic analysis to identify, describe, and analyse themes and patterns within the data.^14^ First, transcripts were read and re-read to familiarise ourselves with the data and identify initial codes of interest. Second, the initial codes were sorted into potential themes, and relevant codes were collated under these themes. Third, themes were reviewed in relation to the generated codes and the entire data set. Finally, themes were named and defined. nvivo 9 was used to organise the codes and themes. For this report, direct quotes are referred to by participant codes to ensure anonymity (participant number; mother/father; V, vaginal birth; and C/S, caesarean section).

## Results

Of the 123 couples or single parents sent a letter of invitation, 39 returned cards indicating an interest in the research (32 mothers and seven fathers). All fathers who took part were married to, or cohabiting with, a mother who also took part in the research. Hence, when ‘couples’ are referred to this indicates 14 participants (seven men and seven women). Men and women in couples were interviewed separately, with the exception of two couples that asked to be interviewed together. Interviews were either in a quiet room in the hospital (*n* = 5) or at the participant's home (*n* = 34). Participants were between 25 and 44 years old, most were white European, and they were either married or co-habiting (see Table 1). The mean gestation at birth was 29.3 weeks. The babies of two mothers died after birth. Participants were recruited from hospital 1 (*n* = 24) and hospital 2 (*n* = 15), but not from hospital 3 (*n* = 0).

**Table 1:** Demographic and obstetric characteristics of the sample

Parent details	*n* = 39 (%)
**Ethnicity**	
White European	29 (74)
Indian	3 (8)
Pakistani	2 (5)
Filipino	2 (5)
Other	3 (8)
**Marital status**	
Married/living with partner	37 (94)
Partner	1 (3)
Separated	1 (3)
**Education**	
None	2 (5)
GCSEs/O levels	9 (23)
A levels/Diploma/City & Guilds	12 (31)
Undergraduate	6 (15)
Postgraduate	2 (5)
Professional	8 (21)
**Employed**	33 (85)
**Income (*n* = 37)**	
<£10 000	3 (8)
£10 000–19 999	7 (19)
£20 000–29 999	15 (41)
£30 000–39 999	6 (16)
>£40 000	6 (16)
	
**Birth details**	***n* = 32 (%)**
	
**Gestation at birth (weeks)**	
31–32	11 (35)
30–31	3 (9)
29–30	3 (9)
28–29	3 (9)
27–28	4 (13)
26–27	4 (13)
25–26	1 (3)
24–25	3 (9)
**Type of birth**	
Vaginal	13 (40)
Caesarean	19 (60)
**Multiple Birth**	11 (34)
**Parity**	
1	24 (75)
2	6 (19)
3	2 (6)

### Overall satisfaction with care

Participants were asked ‘Overall, how satisfied would you say you were with the care that you received during the birth?’ Thirty-one parents (80%) indicated that they were extremely satisfied with their care, and said that there was nothing that could be improved.

Seven participants (18%) indicated that although they were generally satisfied with the care certain things could have been improved, such as the provision of information.

In addition, one woman (2%) was clearly dissatisfied with her care.

### Factors associated with parents' experiences of care

Four main themes emerged as important determinants of either positive or negative experiences of care during preterm birth. Table S1 displays an overview of the themes, with quotes, and shows how many participants mentioned these themes. Frequencies are also shown for women and couples who were interviewed, and these are broadly similar, with the exception of the subtheme of staff appearing confident and in control, which was not mentioned by couples.

### Staff professionalism

This refers to the professional competencies that were important in determining parents' experiences of care. Positive experiences of care were associated with information and explanation, staff being calm in a crisis, and staff appearing confident and in control. In contrast, negative experiences of care were associated with staff being perceived as not listening to the woman.

#### Information and explanation

Provision of information was really important and was mentioned by 33 participants (85%). They wanted to be told what would happen during the birth (particularly if they were having a caesarean section), what type of anaesthetic would be administered, and what was going to happen to their baby when he or she was born. The anaesthetist was someone who stood out in participants' minds in terms of providing detailed information and explanations (1, Mother, C/S; Table S1). It was perceived that someone taking the time to explain what was happening helped them cope with the situation and made the experience less ‘traumatic’ (2, Father, C/S; Table S1). Participants also wanted information to be explained in a way they could easily understand (7, Mother, C/S; Table S1).

One mother wanted more information than she was given during the birth. She had some medical knowledge, and would have liked to know about what was happening throughout her operation in more detail (8, Mother, C/S; Table S1). There can be a lot of people present when a very preterm baby is born, which can be daunting for the parents. However, six participants (15%) commented that the different members of staff introduced themselves and told them what they would be doing. This helped them feel less like they were in a room with people they did not know (6, Mother, C/S; Table S1).

#### Staff calm in a crisis

Nineteen participants (49%) described feeling frightened of what was going to happen during the birth and for the outcome of their baby. However, the calm attitude of the staff helped them feel more comfortable and at ease. For one woman this was the most important factor in her birth experience (19, Mother, C/S; Table S1).

#### Confident and in control

The confidence displayed by staff was important to participants as it demonstrated capability and control. One woman described that the surgeon in charge of her operation portrayed total confidence (5, Mother, V; Table S1). Having confidence in the staff seemed to make it easier to hand over control to them. One woman described that she did not feel that she needed to be in control. She trusted the staff and was happy for them to take control of the situation (5, Mother, V; Table S1). Four mothers (10%) described the doctors as being firm with them, but said this was exactly what they needed. They wanted the staff to take control of the situation and tell them what to do (3, Mother, V; Table S1). The frequencies in Table S1 show that this subtheme was not mentioned by any of the couples interviewed, only by women whose partners did not take part in the study.

#### Staff not listening to the woman

In contrast to most of the other subthemes identified, this is one area that contributed to a negative experience of care for participants. Seven mothers (18%) expressed disappointment that the staff did not always listen to what they had to say. These women described telling staff that they felt they were in labour and close to giving birth, and often the staff did not believe or trust what they were saying, which left women feeling ignored and frustrated. One woman described how she tried to tell the midwife that she was about to have her baby, but was not listened to, and as a result no staff were present at the birth (23, Mother, V; Table S1).

### Staff empathy

Participants' experiences of their care during the birth were also influenced by the interpersonal interactions with care providers, in particular by caring and emotional support, and encouragement and reassurance.

#### Caring and emotional support

Twenty-one participants (54%) spoke about the ‘warm and friendly’ attitude of the staff. In terms of satisfaction with their experience it was important that they were treated in a pleasant manner. The two very different quotes shown in Table S1 (3, Mother, V; 30, Mother, V) illustrate the importance of the staff treating them as an individual and receiving personalised care.

Mothers spoke about the importance of a member of staff always being with them, and this generally referred to the presence of a midwife (2, Mother, C/S; Table S1). One mother whose baby was born with many complications and died less than 24 hours after the birth described how the caring and supportive attitude of one midwife made her experience of the birth less traumatic than it could have been (32, Mother, V; Table S1).

#### Encouragement and reassurance

Twenty-three participants (59%) mentioned wanting encouragement and reassurance from the staff. They understood that staff have to be realistic about the situation and the prognosis for their baby (14, Mother, C/S; Table S1), but found it really helpful and encouraging if the staff were able to reassure them in some way (1, Father, C/S; Table S1). Encouragement from the staff also influenced their experience with care at birth. One woman who was feeling scared and tired described how a midwife encouraged her to continue (3, Mother, V; Table S1). Another mother described how praise from a midwife contributed positively to her experience (23, Mother, V; Table S1).

### Involvement of the father

It was important to the mothers that the baby's father was involved in the birth, and the extent to which staff involved them contributed to a positive or negative experience with care. For example, two women (5%) described how the staff tried to delay the caesarean section so the father could get there for the birth. Three women (8%) also discussed that they had planned their partner's involvement in the birth, and therefore appreciated any effort the staff made to make them feel more involved (2, Mother, C/S; Table S1). Four women (10%) talked of regret that the baby's father was not able to participate more and was not encouraged to feel more involved in the birth by the staff (5, Mother, V; 31, Mother, V; Table S1).

It was also important to fathers that they were encouraged to feel involved in the birth. One of the fathers interviewed described how fathers are not normally made to feel involved in the birth, but that this time he was involved from the start (2, Father, C/S; Table S1).

### Birth environment

Participants discussed features of the delivery suite and operating theatre that contributed to their positive experience at the birth. Five participants (13%) described that the radio was playing during the birth, which made the environment seem less frightening (1, Mother, C/S; Table S1). Three women (8%) also commented on the views from the windows of the operating theatre. It helped them feel ‘connected’ with the outside world and help take their mind off things (14, Mother, C/S; Table S1).

## Discussion

The aim of this study was to explore parents' experiences and satisfaction with care during very preterm births. Overall, parents' experiences of care were positive, with only one mother expressing dissatisfaction with the care she received during the birth. This is reassuring, and typical of studies of satisfaction with maternity services generally.^15^,^16^ Our study identified four dimensions of care that are important determinants of parents' experiences of care during the birth of their preterm baby: staff professionalism, staff empathy, involvement of the father, and the birth environment.

Information provision and emotional support from the staff during the birth are clearly important to parents. Parents were highly satisfied with the information they were given during the birth, which is in contrast to previous studies where mothers of preterm babies have perceived the staff as not having the time to give information before or during the delivery.^17^,^18^ The need to feel supported by professionals is widely recognised, and has been shown to be a key factor in women's satisfaction with maternity care more generally.^19^–^22^ The stress and uncertainty surrounding preterm birth are likely to increase the need for emotional support and reassurance from the staff.^23^

Overall, the determinants of parents' experiences with care were generally consistent with previous research on term births; however, two factors unique to preterm birth were identified. These were the importance of the staff appearing calm during the birth and staff taking control during the birth. A calm response shown by the staff has not been previously identified as associated with satisfaction with care during childbirth, although it has been identified in other areas of patient satisfaction with care, particularly relating to emergency situations.^24^ Being calm during an emergency in maternity units has also been reported by health professionals as a factor in promoting effective teamwork.^25^ During preterm birth, parents are often distressed and in shock; therefore, a calm response by the staff may help them deal with the difficult situation. In contrast, research into satisfaction following term birth emphasises the importance of the woman being in control and involved in decision making in her birth.^9^,^26^,^27^ Some previous studies have suggested that the role of control may be different in complicated childbirths.^28^ Therefore, during the birth of a very preterm baby it seems to be important to some parents that the staff take control of the situation and make decisions quickly. Although it's important to note that in this study staff being in control was only mentioned by women whose partners did not take part, but not by the couples interviewed.

Although parents largely reported positive experiences of care, two areas emerged where parents felt things could have been improved. First, some women described being in labour or very close to giving birth, and the staff either not believing them or not appearing to listen to what they said. Similar experiences were reported in a national study of women's experiences of maternity care.^21^ The importance of listening to mothers in preterm labour is paramount. Second, parents valued feeling that the father was involved in the birth. Some fathers described feeling marginalised. This is consistent with a previous study that suggested that fathers of preterm infants often feel like they are considered to be secondary to mothers by members of the healthcare team.^29^ Involvement of the father during preterm birth is important as it can facilitate father–infant attachment.^30^ This is especially relevant as the baby will be admitted to the neonatal unit, which can disrupt the normal attachment process between parent and child.^31^ Also, fathers are an additional source of support to the mother during the birth. Continuous support is associated with positive outcomes and enhanced satisfaction with the birth experience in term births.^32^ Finally, there is an increasing emphasis on the importance of family-centred care in neonatal units, which emphasises the involvement and inclusion of both the mother and the father.^33^ This study suggests that family-centred care should extend, wherever possible, to the birth of the baby.

In common with other studies of satisfaction with care, one possible limitation of this study is that parents may have been reluctant to criticise the professionals who have taken care of them and their preterm baby. This ‘halo effect’ may be even more pronounced in the parents of preterm babies as the staff have looked after their baby for many weeks.^15^ Similarly, some researchers raise the issue that women do not know what care during birth *should* be like, and therefore just evaluate the status quo.^16^,^34^ An advantage of our study was the use of in-depth interviews by a researcher not associated with the hospital, which should have been more able to pick up negative experiences compared with questionnaires. Trustworthiness was also enhanced by the careful construction of the interview questions, the use of a well-established and appropriate form of analysis, ensuring that participants were given adequate opportunity to refuse participation in the study, the encouragement of a rapport between interviewer and interviewee, frequent debriefing sessions between the team members, and a discussion of results with peers who were not part of the research team.

One-quarter of parents accepted the invitation to be interviewed, which is a good response for this type of study. However, the experiences reported in this study may not be applicable to all parents who have a very premature baby. For example, the sample mainly consisted of white, married women, and further research is needed to see how these experiences relate to parents from different backgrounds. The implications and conclusions of this study should be considered with this in mind.

In spite of these limitations, this study provides a rich and detailed insight into parents' experiences with care during preterm birth, and the findings have important implications for practice. Provision of information and explanations to parents, and offering caring and emotional support, is extremely important to parents. The parents in this study were very positive about these aspects of care during the birth; however, the findings also suggest that women should be listened to more, especially when telling staff they were in labour or felt close to giving birth, and that fathers should be helped to feel involved in the birth. The experience of very preterm birth is different to a term birth, and existing questionnaire measures of satisfaction with care in parents of term babies may not be appropriate for this population of parents. Satisfaction with care is a multidimensional construct consisting of different components of care provision that affect satisfaction. Therefore, it is important that future studies include multidimensional measures of satisfaction with care, as single-item measures only provide a limited insight into parents' experiences.

## Conclusion

In summary, this study contributes to the limited literature on satisfaction with care during very preterm birth. Giving birth to a preterm baby is a distressing and traumatic time for most parents. In spite of this, the present study suggests that parents are very satisfied with the care provided during the birth, but that listening to women and involving fathers during the birth could increase this further.

## Supporting Information

**Table S1.** Themes, quotes, and number of interviews in which themes were mentioned.
