# Supplementary material for: Parents' experiences and satisfaction with care during the birth of their very preterm baby: a qualitative study
Source: BJOG. 2013 Jan 4;120(5):637–43. doi: 10.1111/1471-0528.12104 (PMC3613739; doi:10.1111/1471-0528.12104)
Supplement: Supplementary file 1 [file bjo0120-0637-SD1.docx]

**Table S1. Themes, quotes, and number of interviews in which themes were mentioned**

| **THEME** | **ILLUSTRATIVE QUOTES** | **INTERVIEWS** | | | |
| --- | --- | --- | --- | --- | --- |
|  |  | **All mothers**  (n = 32) | **Couples** | | **Total**  **(N = 39)** |
|  |  |  | Father  (n = 7) | Mother  (n =7) |  |
| **Staff Professionalism** |  |  |  |  |  |
| Information and explanation | *“so we actually go down into the operating theatre and again the anaesthesiologist was there and talking to [us] as she said ‘I will stay with you the whole time’....and she talked us through everything that was happening and for both of us that was just outstanding, absolutely”* (1 Mother, C/S).  *“it was a traumatic experience. I think, if it hadn’t been explained to us exactly step by step it would have been more traumatic...It was just so much easier, because they did go out of their way and they explained absolutely everything to you”* (2 Father, C/S).  *“They told you everything that was going on, what was happening. They make sure you understood, make sure he [father] understood what was going on” (*7 Mother, C/S*).*  *“So you feel prodding, and I wasn’t told much. I felt I wasn’t told much when I was actually in there and hadn’t, I didn’t know when they’d started to open me up, cut me open...So I didn’t know what they were doing, water’s, broken my waters....None of that was ever communicated to me.”* (8 Mother, C/S).  *“I mean they were all very, I remember there being 16 people in the room and they were all introducing themselves and what they did”* (6 Mother, C/S). | 29 | 4 | 6 | 33 |
| Staff calm in a crisis | *“you’re not as frightened. It’s daunting going in a room when you’ve never been in. All your bits are going to be on show. And you’re worried about your children. Are they gonna survive? Are they gonna be born stillborn? You know....they were so relaxed, they made me feel so comfortable”* (4 Mother, C/S).  *“I think it was them staying relaxed. Even though it was a rush, it was a stressful time, you could see that, but they were very good at staying calm. But I suppose that’s their job in a way, but they were actually very good at it”* (19 Mother, C/S). | 10 | 1 | 3 | 11 |
| Confident and in control | *“And the way he mastered the team, I got the absolute… he had an air of confidence and er control of the entire team. He knew what every person was doing. And he was very commanding as well”* (5 Mother, V).  *“Absolute confidence in the staff. I didn’t feel like I needed to know every step of the way. I was able to just step back, realise that control was not mine. The control was where it should be, with professionals, and they would take good care of them [the babies]”* (5 Mother, V).  *“it was very very quick, very shouty: ‘you have to do this, you have to do this now’. It was made very clear to me if I didn’t push he wouldn’t survive. Erm, which was absolutely fantastic, which was what needed to be done”* (3 Mother, V). | 8 | 0 | 0 | 8 |
| Staff not listening to the woman | *“The only kind of downside to it, was I kept saying to her, all my family have very quick labours....... I kept saying to her I need to push I need to push and she said I’ve only checked you half an hour ago, you’re only 3cm ..... and she went I’m just popping out the room.....and at that point I just pushed and her head popped out, and no one was in the room apart from me and my partner”*(23 Mother, V).  *“And then when I started to get pains, I started to tell the midwives, or the nurses that were there. And felt that they didn’t actually believe me, because they put me on monitors. And where my waters had gone, the monitors don’t pick up the contractions as well. So they were just saying ‘no, no, no, the contractions are not real.....basically [you] can’t be feeling this amount of pain”* (19 Mother, C/S). | 7 | 1 | 1 | 8 |
| **Staff Empathy** |  |  |  |  |  |
| Caring and emotional support | *“I just found our experience very good, it was very I suppose personal in a sense. I wasn’t, I didn’t feel like a piece of meat. I felt like a human.....and people were caring”* (3 Mother, V).  *“But the midwives that should have shown me compassion in the beginning didn’t. They were just not bothered”* (30 Mother, V).  *“one of the nurses just steps out the way, holds your hand, and talks to you.....So it’s just nice to have someone there, talking to you and holding your hand and sort of walking you through everything instead of everyone buzzing around”* (2 Mother, C/S).  *“the midwives were incredible, so during the birth,...we had this amazingly lovely kind of West African um midwife who was, oh just love, like lovely, so nice so, supportive and caring and empathetic and everything that you could possibly want and just really supportive and, so the birth process itself actually, in the scheme of things was relatively easy thing then to go to because I felt very supportive... and she was so lovely”* (32 Mother, V). | 20 | 1 | 5 | 21 |
| Encouragement and reassurance | *“Obviously so they can’t lie... but just kind of being positive I think really really helps um ‘cause you know, it’s it’s quite terrifying not having had an operation before and um you know you don’t quite know what to expect and things so just people you know just reassuring you, saying nice things”* (14, C/S Mother).  *“And that’s what you want is reassurance, that time, and so yeah, it was very good”* (1 Father, C/S)”.  *“Yeah we were whisked upstairs and at that point I couldn’t feel the hand moving so I really freaked out. One of the midwives was there and she could feel a pulse, calm down, gave me cuddles, really calmed me down and said ‘you’re ok, you’ve got to do this, you’ll get through it.’ Really sort of geed me up and gave me that extra bit of strength really*” (3 Mother, V).  *“you know she was constantly praising “you, you’re doing really well, just breathe through it”, you know and things like that whereas you get some midwives who just aren’t the nicest, so um, the fact that she was as nice as she was”* (23 Mother, V). | 20 | 3 | 4 | 23 |
| **Involvement of Father** | *“He got there really quick. But they involved him, once they brought him [to the operating theatre], they told him everything while he was getting changed, what to expect.”* (2 Mother, C/S).  *“I found it reassuring that they were very happy with [husband] to be sort of looking over their shoulders and sticking his nose in and whatever, so there was no “stand over there dad”* (12 Mother, C/S).  *“Erm he found it very awkward...When they were being born he just sat out there, wasn’t really able to participate...So he felt like a spare part......when we were rushed to the surgical unit… there were so many people in the room, he felt he didn’t know where to stand. He didn’t want to get in the way. He knew he needed to get there..let everyone get on with their job. But he felt in the way”* (5 Mother, V).  *“I don’t think anyone even really spoke to [the father], I mean I I’m reflecting on it now, I don’t think anyone did, how was he involved, he wasn’t involved at all, so yeah how are you feeling, is there anything I can do, yeah”* (31 Mother, V).  *“Because normally they don’t talk to you. To a woman, they say ‘right we’ve got to do this, got to do that’ so the lady knows exactly what’s happening to her and why. For the bloke......‘Stay down the pub and we’ll give you a ring when it’s all done and you can come up when it’s all nice and clean, in a blanket.’ But with [name of hospital], it was completely different”* (2 Father, C/S). | 11 | 5 | 4 | 16 |
| **Birth Environment** | *“you know they didn’t make it scary in any way at all, they were all quite happy, I think the radio was playing, which was good, you know things like that. The environment didn’t seem scary”* (1 Mother, C/S).  *“it can take your mind off it a bit rather than just sort of grey walls um so yea so I mean that’s very much what we remember actually and often sort of comment on it you know to people”*  (14 Mother, C/S). | 14 | 3 | 3 | 17 |
